# Preventive Effect of YGDEY from Tilapia Fish Skin Gelatin Hydrolysates against Alcohol-Induced Damage in HepG2 Cells through ROS-Mediated Signaling Pathways

**DOI:** 10.3390/nu11020392

**Published:** 2019-02-13

**Authors:** Mei-Fang Chen, Fang Gong, Yuan Yuan Zhang, Chengyong Li, Chunxia Zhou, Pengzhi Hong, Shengli Sun, Zhong-Ji Qian

**Affiliations:** 1 College of Food Science and Technology, Guangdong Ocean University, Zhanjiang 524088, China; meifangchen93@163.com (M.-F.C.); m13025612271@163.com (F.G.); zyyla92@126.com (Y.Y.Z.); chunxia.zhou@163.com (C.Z.); hongpengzhigdou@163.com (P.H.); 2 School of Chemistry and Environment, Guangdong Ocean University, Zhanjiang 524088, China; cyli_ocean@163.com (C.L.); xinglsun@126.com (S.S.); 3 Shenzhen Institute of Guangdong Ocean University, Shenzhen 518114, China

**Keywords:** YGDEY, Alcohol metabolism, Oxidative stress, HepG2 cells, DNA damage

## Abstract

According to a previous study, YGDEY from tilapia fish skin gelatin hydrolysates has strong free radical scavenging activity. In the present study, the protective effect of YGDEY against oxidative stress induced by ethanol in HepG2 cells was investigated. First, cells were incubated with YGDEY (10, 20, 50, and 100 μM) to assess cytotoxicity, and there was no significant change in cell viability. Next, it was established that YGDEY decreased the production of reactive oxygen species (ROS). Western blot results indicated that YGDEY increased the levels of superoxide dismutase (SOD) and glutathione (GSH) and decreased the expression of gamma-glutamyltransferase (GGT) in HepG2 cells. It was then revealed that YGDEY markedly reduced the expressions of bax and cleaved-caspase-3 (c-caspase-3); inhibited phosphorylation of Akt, IκB-α, p65, and p38; and increased the level of bcl-2. Moreover, the comet assay showed that YGDEY effectively decreased the amount of ethanol-induced DNA damage. Thus, YGDEY protected HepG2 cells from alcohol-induced injury by inhibiting oxidative stress, and this may be associated with the Akt/nuclear factor-κB (NF-κB)/mitogen-activated protein kinase (MAPK) signal transduction pathways. These results demonstrate that YGDEY from tilapia fish skin gelatin hydrolysates protects HepG2 cells from oxidative stress, making it a potential functional food ingredient.

## 1. Introduction

Alcohol is primarily metabolized in the liver [1], and the adverse effects of ethanol metabolism have been observed largely [2] through the increased formation of reactive oxygen species (ROS), including the superoxide radical (O^2−^), the hydroxyl radical (OH•), and hydrogen peroxide (H_2_O_2_). During normal metabolic processes, ROS are effectively eliminated by the human body’s antioxidant defense systems, such as the enzymes superoxide dismutase (SOD) and glutathione (GSH) [3]. However, pathological conditions are often associated with oxidative stress in numerous chronic diseases [4]. Oxidative stress induces lipid peroxidation, apoptosis of hepatocytes, and damage to biological membranes, proteins, and DNA [5]. Apoptosis can be activated by proteins in the bcl-2 family, the cysteinyl aspartate-specific proteinase (caspase) family, and other signaling molecules, such as Akt, nuclear factor-κB (NF-κB), and mitogen-activated protein kinases (MAPKs) [3,6,7]. Oxidative stress has been detected in a variety of liver diseases, including alcoholic liver disease (ALD), non-alcoholic fatty liver disease (NAFLD), and hepatocellular carcinoma [8]. ALD, a major health problem, is primarily caused by the byproducts of alcohol metabolism, and oxidative stress is a primary causal factor of alcohol-induced liver injury. This is especially the case when the liver has lower levels of antioxidants to counteract the generation of ROS. It has been established that oxidative stress is related to the pathogenesis of ALD, as ROS generation has been observed in alcohol-exposed cultured cells and alcohol-exposed mouse embryos [3].

Oxidative stress and antioxidants have been extensively investigated in many recent research endeavors [9]. The imbalance between the generation and the elimination of ROS leads to oxidative stress, ultimately causing cell death. Oxidative stress has been linked to many health disorders, such as inflammation, neurodegenerative and cardiovascular diseases, and cancer [10]. Antioxidants may have protective effects against oxidative damage in humans by reducing the formation of ROS [11]. Recently, a series of studies found that radical scavengers can protect the human body from oxidative stress. For instance, genistein ameliorates alcohol-induced liver injury by reducing oxidative stress [12]. As a result of the high toxicity commonly experienced with conventional chemotherapy, many natural products have been used as alternative treatments for diseases [13]. Many antioxidant compounds, especially fish peptides, have been widely studied. Bioactive peptides have been isolated from *Oreochromis niloticus* skin gelatin hydrolysates and tilapia frame and skin enzymatic protein hydrolysates [14,15]. Protein hydrolysates and peptides exhibit many physiological functions, such as antimicrobial, antioxidant, antithrombotic, antihypertensive, and immunomodulatory activities [16]. A peptide’s activity is closely associated with its size, amino acid composition, sequence, and particularly the hydrophobicity of its constituent amino acids. Numerous studies have reported that peptides derived from the gelatin hydrolysates of a variety of fish species (salmon, trout, tuna, and tilapia) possess antioxidant and antihypertensive properties [17].

Tilapia, an important fish species in freshwater aquaculture [18], has become one of the leading industries of agricultural aquaculture in China [19]. It is the second most cultured fish after carps [20]. Its commercial value is the result of a high growth rate, increased disease resistance, ease of cultivation under controlled conditions, resistance to environmental stress, and acceptance by consumers [21]. In the global market, the demand for tilapia in all forms is increasing rapidly. It is usually processed into fillets accompanied by a large number of byproducts, such as skin, scales, bones, etc. [15]. Most of the byproducts are considered waste; however, a significant amount of protein remains in these byproducts with several nutritional benefits, including a good array of essential amino acids. Fish skin, in particular, is a rich source of collagen and gelatin, which can be used as food ingredients to provide elasticity and viscosity; it has also found use in medical applications [22]. Tilapia skin collagen peptide, a bioactive peptide, was observed to have antioxidant activity in mice [23]. NPALATEPDPMPF (1382.57 Da) from Nile tilapia (*Oreochromis niloticus*) scale gelatin mitigates oxidative stress and has potential use as a functional food ingredient [9]. These peptides have different activities, such as antioxidant and anti-apoptotic effects and differentiation induction. Heating collagen to at least 45 °C converts it into gelatin, which is then acquired for further use [14]. Recently, it was reported that fish skin gelatin is a good substrate for enzymatic hydrolysis to produce bioactive peptides [14]. These bioactive molecules are short peptides of only 3–20 amino acid residues [17]. Some fish hydrolysates have been found to have noticeable antioxidant, antihypertensive, antimicrobial, immuno-modulatory, and cholesterol-lowering effects [9]. For example, peptides (VGLPNSR, 741.4133 Da; QAGLSPVR, 826.4661 Da) with angiotensin I-converting enzyme inhibitory activity have been identified from tilapia skin gelatin hydrolysates [18]. Four antibacterial peptides from the protamex hydrolysate of Atlantic mackerel (*Scomber scombrus*) byproducts have been purified and characterized [24]. Furthermore, previous studies have indicated that tilapia fish skin gelatin hydrolysates suppress photoaging in vivo and that LSGYGP exhibits strong hydroxyl radical scavenging activity [25]. Zhang [26] found that YGDEY from the enzymatic hydrolysates of tilapia (*Oreochromis niloticus*) skin gelatin had high hydroxyl radical scavenging activity. However, the antioxidant activity of YGDEY in human hepatoma cells (HepG2 cell line) is yet to be examined. The findings from this study may provide a basis for further research.

In the present study, we investigated the protective effects of YGDEY from tilapia fish skin gelatin hydrolysates against ethanol-induced HepG2 cell injury. The aims of studying HepG2 cells treated with YGDEY are to evaluate this peptide’s (1) effects on the viability of HepG2 cells; (2) effects on the generation of intracellular ROS; (3) effects on DNA damage in HepG2; (4) activation or inhibition of SOD, GSH, and gamma-glutamyltransferase (GGT) in HepG2 cells; and (5) effects on the activation of cell signaling proteins. The results show that YGDEY could be a novel therapeutic agent against ethanol-induced oxidative stress in HepG2 cells and that its protective effects are related to antioxidant enzymes and AKT/NF-κB/MAPK signaling pathways.

## 2. Materials and Methods

### 2.1. Materials and Reagents

YGDEY with a 99.11% purity was purchased from Hangzhou Dangang Biotechnology Co., Ltd. (Hangzhou, China). The human hepatoma cell line (HepG2) was provided by Cell Bank of the Chinese Academy of Sciences (Shanghai, China). Dulbecco’s modified Eagle’s medium (DMEM) from HyClone was used. Fetal bovine serum (FBS), trypsin-EDTA (0.25%), and penicillin/streptomycin were purchased from Gibco (New York, NY, USA). 3-(4,5-Dimethylthiazol-2-yl)-2,5-diphenyltetrazolium bromide (MTT), dimethyl sulfoxide (DMSO), 2’,7’-dichlorodihydrofluorescein diacetate (DCFH-DA), and 4’,6-diamidino-2-phenylindole (DAPI) were purchased from Sigma-Aldrich (Steinheim, Germany). The BCA protein assay kit was provided by Thermo Scientific, USA. The following were purchased from Santa Cruz Biotechnology Inc. (Santa Cruz, CA, USA): Mouse monoclonal antibodies, including β-Actin (130300), GGT (100746), SOD (271014), GSH (71155), bcl-2 (7382), bax (20067), caspase-3 (7272), IκB-α (1643), p-IκB-α (8404), NF-κB p65 (8008), p-NF-κB p65 (136548), NF-κB p50 (8414), p-NF-κB p50 (271908), JNK (7345), p-JNK (6254), p-ERK (81492), and p-p38 (166182); rabbit polyclonal antibodies (ERK, sc-94; p38, sc-535); and secondary antibodies (goat anti-mouse IgG-HRP, sc-2005; goat anti-rabbit IgG-HRP, sc-2004). Akt (9272), cleaved-caspase-3 (9661), and phospho-Akt (4060) rabbit antibodies were provided by Cell Signaling Technology (CST, USA). All chemicals were of analytical grade.

### 2.2. Cell Culture

HepG2 cells were maintained in DMEM and supplemented with FBS (10%) and penicillin/streptomycin (1%). Cells were cultured in a CO_2_ incubator (37 °C, 5% CO_2_) and used in assays before reaching 80% confluence.

### 2.3. Cell Viability Assay

HepG2 cells (2 × 10^4^ cells/well) were seeded and incubated (37 °C, 5% CO_2_, 24 h). HepG2 cells were added to ethanol (0, 0.25, 0.5, 0.75, 1, 1.5, 1.75, and 2 M). At the same time, cells were incubated with YGDEY (10, 20, 50, and 100 μM). After 24 h incubation, cell viability was assessed using the MTT method. The cells were incubated with MTT (1 mg/mL, 200 μL) at 37 °C in a CO_2_ incubator (5% CO_2_) for 4 h. Subsequently, DMSO (200 µL) was added. The absorbance of each well was detected using a microplate reader (BioTek, Winooski, VT, USA) at 570 nm.

### 2.4. Cell ROS Determination by DCFH-DA

Intracellular formation of ROS was determined via the fluorescent probe DCFH-DA. Cells were plated in 24-well plates, and YGDEY (10, 20, 50, and 100 μM) was added to the wells. After 2 h, cells were exposed to 0.75 M ethanol. DCFH-DA (10 μM, 200 μL) was added to each well. After reacting for 20 min at 37 °C in the dark, cells were washed with phosphate buffer saline (PBS). The formation of 2’,7’-dichlorofluorescein (DCF), due to the oxidation of DCFH-DA via intracellular ROS, was immediately examined using an inverted fluorescence microscope (Olympus Opticals, Tokyo, Japan).

### 2.5. Western Blot Analysis

The treated cells were lysed in RIPA lysis buffer containing 1% phenylmethanesulfonyl fluoride (PMSF). The samples were centrifuged (4 °C, 12,000 rpm, 20 min), and the protein concentration of the supernatant was quantified using the BCA protein assay kit. The samples were subjected to 10% SDS-PAGE and transferred to NC membranes. The membranes were activated with ddH_2_O and blocked with 5% non-fat milk in TBST for 4 h. The membranes were incubated with primary antibodies (1:400 in TBST) overnight at 4 °C, after which they were washed four times (10 min each wash) with TBST. The membranes were incubated with secondary antibodies (1:4000) for 2 h and then washed four times with TBST. The membranes were visualized with an enhanced chemiluminescence (ECL) detection system (Syngene, Cambridge, UK).

### 2.6. Immunofluorescence Assay

Cells (5 × 10^3^ cells/well) were treated as described above, fixed using 4% paraformaldehyde (4 °C, 30 min), and then permeabilized for 10 min with PBS containing 0.2% Triton X-100. Bovine serum albumin (BSA; 5%) was added to each well, and blocking occurred for 1 h. Cells were incubated with antibodies specific for NF-κB p65 (1:75) diluted in 1% BSA overnight at 4 °C. After three washes with PBS, the cells were incubated with goat anti-mouse IgG Dylight 488 (1:500) for 2 h at room temperature. Cells were stained with DAPI (100 ng/mL, 5 min). Images were observed using an inverted fluorescence microscope with blue and green fluorescence (magnification: 10×).

### 2.7. Comet Assay

DNA damage was evaluated by single cell gel electrophoresis (comet assay) [27]. Cells were treated as described above. Cells were rinsed three times with PBS (0.01 M, pH 7.4), detached by EDTA-trypsin, and suspended in PBS (1 × 10^5^ cells/mL). A solution of 0.8% normal agarose (NMA, 100 μL) dissolved in PBS was dropped gently onto a microslide, covered immediately with a coverslip, and then placed at 4 °C for 15 min. The coverslip was removed after the gel had formed. The cell suspension (200 cells/μL, 20 μL) was combined with 1% low melting point agarose (LMA, 80 μL) preserved at 40 °C, mixed gently by pipetting up and down, coated over the microslide, covered immediately with a coverslip, and then placed at 4 °C for 15 min. The coverslip was again removed after the gel had solidified. The slides were immersed in an ice-cold lysis solution (LS: 2.5 M NaCl, 100 mM Na_2_EDTA, 10 mM Tris, 200 mM NaOH, pH 10, 1% sodium lauryl sarcosinate, and 1% Triton X-100) at 4 °C for 90 min. The slides were washed thrice with ice-cold ddH_2_O, and then gently immersed in alkaline electrophoresis solution (AES: 200 mM NaOH, 1 mM Na_2_EDTA, pH > 13) to initiate DNA unwinding (4 °C, 0.5 h). Then, electrophoresis was performed (25 V, 20 min). After electrophoresis, the slides were gently immersed in PBS. Cells were stained with DAPI (50 μg/mL, 20 μL) in the dark for 5 min. The slides were rinsed briefly with ddH_2_O, dried completely at 37 °C, and observed using fluorescence microscopy.

### 2.8. Molecular Docking Analysis

The three-dimensional (3D) crystal structure of bcl-2 (ID: 4IEH) [28] was selected from the Protein Data Bank (PDB) (http://www.rcsb.org/pdb/). The structure of YGDEY was designed with ChemProfessional 15.0 (v15.0.0.106 Chemdraw, PerkinElmer Informatics, MA, USA). To simulate the protein–ligand interaction, the CDOCKER algorithm in Discovery Studio (DS) 3.5 (Accelrys Software Inc., San Diego, CA, USA) was used. The high-molecular dynamics method was utilized to randomly search for small molecule (YGDEY) conformations, and simulated annealing was used to optimize each conformation of the receptor’s (bcl-2) active site.

### 2.9. Statistical Analysis

All data were analyzed by one-way ANOVA accompanied by Dunnett’s Multiple Comparison Test for group comparison. Data are expressed as the mean ± SD (*n* = 3). GraphPad Prism 5 (GraphPad Prism Software Inc., La Jolla, CA, USA), Image J (Version 1.46r, NIH), and comet assay software project (CASP Version 1.2.3 beta1, Krzysztof Konca, CaspLab.com) were used for data analysis.

## 3. Results

### 3.1. Effect of YGDEY on Cell Viability of HepG2 Cells

**Figure 1:**
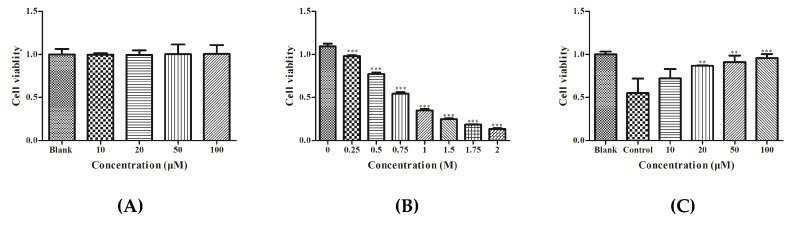
(**A**) The cytotoxic effects of YGDEY on HepG2 cells. Cells were co-cultured with YGDEY (10, 20, 50, and 100 μM) for 24 h, and cell viability was evaluated by MTT assay. Data are shown as means ± SD (*n* = 3). (**B**) Cell viability of ethanol-induced HepG2 cells. Cells were treated with ethanol of different concentrations (0, 0.25, 0.5, 0.75, 1, 1.5, 1.75, and 2 M) for 24 h. Cell viability was evaluated by MTT assay. Data are shown as means ± SD (*n* = 3). *** compared with the no-alcohol blank group, *p* < 0.001. (**C**) Protective effects of YGDEY in HepG2 cells. Cells were pretreated with YGDEY for 24 h prior to treatment with 0.75 M ethanol for 2 h. After the treatment, cell viability was evaluated by MTT assay. Data are shown as means ± SD (*n* = 3). ** compared with the control group, *p* < 0.01. *** compared with the control group, *p* < 0.001.

HepG2 cells were treated with different concentrations of YGDEY (10, 20, 50, and 100 μM). The MTT assay results show that YGDEY did not have a cytotoxic effect on HepG2 cells (Figure 1A). Figure 1B shows that ethanol decreased cell viability in a dose-dependent manner. Cell viability was approximately 50% when cells were exposed to 0.75 M ethanol. As depicted in Figure 1C, treatment with YGDEY significantly increased the viability of HepG2 cells induced by 0.75 M ethanol.

### 3.2. ROS Production in HepG2 Cells

**Figure 2:**
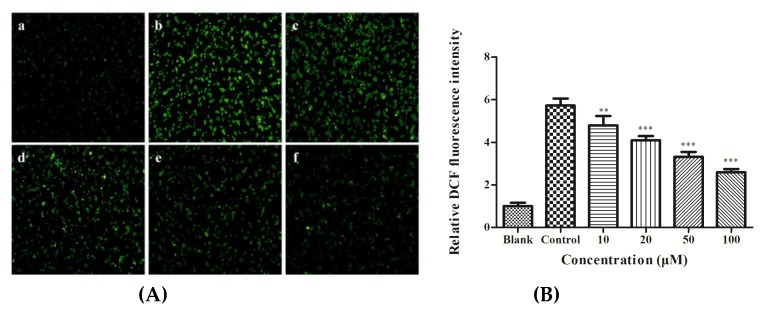
Effect of YGDEY on the intracellular reactive oxygen species (ROS) level. (**A**) HepG2 cells were pretreated with YGDEY for 24 h before treatment with 0.75 M ethanol for 2 h. Then, the cells were exposed to DCFH-DA for 20 min. DCF fluorescence of the treated cells was measured by using an inverted fluorescence microscope. (a) HepG2 cells without treatment (the blank group); (b) cells exposed to 0.75 M ethanol (the control group); (c, d, e, and f) cells pretreated with YGDEY (10, 20, 50, and 100 μM, respectively) prior to treatment with 0.75 M ethanol. (**B**) The relative DCF fluorescence intensity. Data are shown as means ± SD (*n* = 3). ** compared with the control group, *p* < 0.01. *** compared with the control group, *p* < 0.001.

The cellular ROS scavenging activities of various concentrations of YGDEY are reported in Figure 2. As depicted in Figure 2A, in the blank group, no obvious fluorescence was observed, while high ROS levels were noted in the control group. Treatment with YGDEY for 24 h decreased the levels of ROS in a dose-dependent manner (Figure 2B). These results demonstrate that YGDEY may protect HepG2 cells from oxidative damage.

### 3.3. Effect of YGDEY on Antioxidant Enzymes SOD, GSH, and GGT

**Figure 3:**
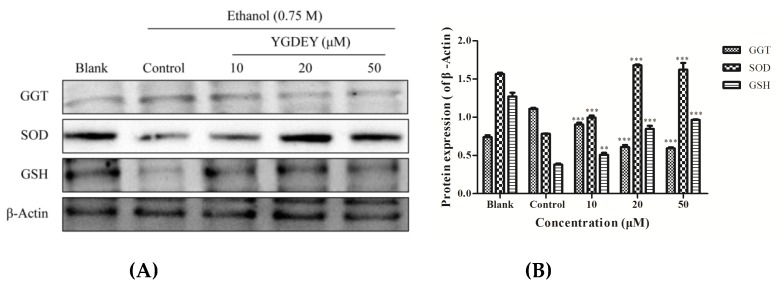
(**A**) Western blot analysis to examine the effect of YGDEY on SOD, GSH, and GGT in ethanol-induced HepG2 cells. YGDEY (10, 20, and 50 μM) was added to cells for 24 h before treatment with 0.75 M ethanol for 2 h. β-Actin was used as an internal control. (**B**) Protein expression (relative to β-Actin) was evaluated. Data are shown as means ± SD (*n* = 3). ** compared with the control group, *p* < 0.01. *** compared with the control group, *p* < 0.001.

SOD is an important scavenger of intracellular ROS [29]. GSH is the main antioxidative protector against oxidative stress in the liver and its role in ALD [30]. GGT is widely used as a marker of excessive alcohol intake in patients with ALD [31]. As depicted in Figure 3, SOD and GSH levels were markedly increased in HepG2 cells treated with YGEDY (10, 20, and 50 μM) compared with the control group. Meanwhile, the GGT level significantly decreased. The results indicate that YGDEY could reduce ethanol-induced oxidative stress in HepG2 cells by regulating key antioxidant enzymes (SOD, GSH, and GGT).

### 3.4. Effect of YGDEY on the Expression of bcl-2, bax, and caspase-3

**Figure 4:**
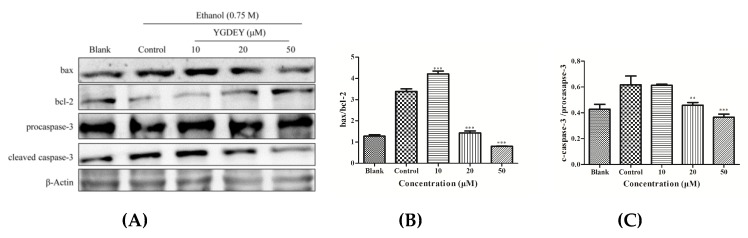
(**A**) The expressions of bax, bcl-2, procaspase-3, and caspase-3 p20 in HepG2 cells. Cells were pretreated with YGDEY (10, 20, and 50 μM) for 24 h before induction by 0.75 M ethanol for 2 h. β-Actin was used as an internal control. (**B**) and (**C**) The ratios of bax/bcl-2 and cleaved-caspase-3/procaspase-3 were calculated. Data are shown as means ± SD (*n* = 3). ** compared with the control group, *p* < 0.01. *** compared with the control group, *p* < 0.001.

As depicted in Figure 4, the expression of bax and cleaved-caspase-3 (c-caspase-3) was greatly increased in ethanol-treated HepG2 cells, while the level of bcl-2 was significantly reduced compared with the blank group. YGDEY treatment reduced the expressions of bax and c-caspase-3 and augmented the level of bcl-2. Compared with the control group, the expression of procaspase-3 did not change. The effect of YGDEY on bcl-2, bax, and c-caspase-3 could be involved in its protective effects against oxidative stress.

### 3.5. Effect of YGDEY on the Activation of Akt and NF κB

**Figure 5:**
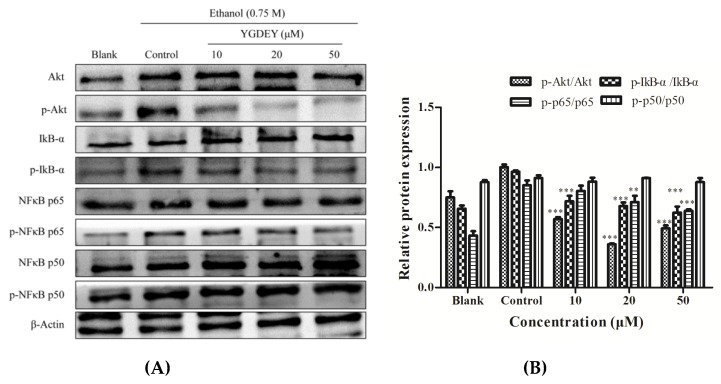
(**A**) Expression changes of phosphor-Akt and phosphor-NF-κB in HepG2 cells. Cells were exposed to YGDEY (10, 20, and 50 μM) for 24 h before treatment with 0.75 M ethanol for 2 h. β-Actin was used as an internal control. (**B**) Relative protein expression was normalized. Data are shown as means ± SD (*n* = 3). ** compared with the control group, *p* < 0.01. *** compared with the control group, *p* < 0.001.

It can be seen from Figure 5 that the phosphorylation of Akt, IκB-α, and NF-κB p65 was remarkably increased in HepG2 cells induced by ethanol compared with the blank. Treatment with YGDEY led to significantly reduced expressions of p-Akt, p-IκB-α, and p-p65. There was no significant difference in p-p50 levels.

### 3.6. Immunofluorescence Analysis

**Figure 6:**
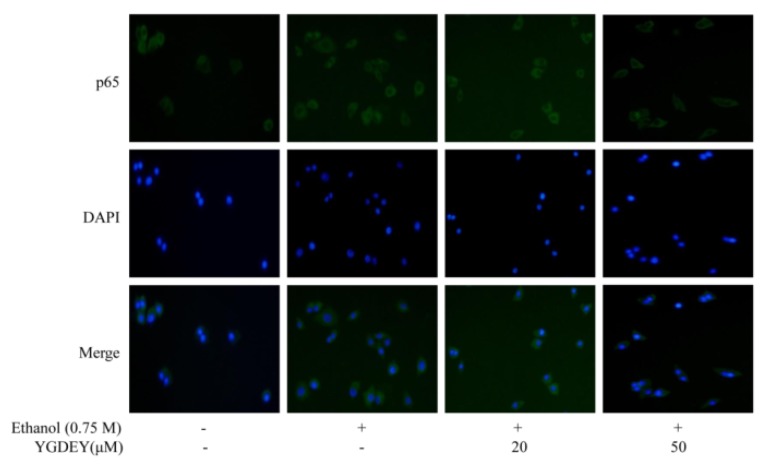
Effect of YGDEY on the cellular localization of p65 in HepG2 cells. Cells were treated with YGDEY (20 and 50 μM) for 24 h prior to ethanol treatment for 2 h. Images were obtained using an inverted fluorescence microscope.

To evaluate whether p65 localization was altered as a result of ethanol-induced HepG2 cell damage, an immunofluorescence assay was conducted by fluorescent microscopy. As shown in Figure 6, after exposure to 0.75 M ethanol for 24 h, HepG2 cells showed significant cytoplasmic immunostaining for p65 compared with the blank group. However, treatment with YGDEY (20 and 50 μM) decreased the translocation of p65 from the cytoplasm to the nucleus. On these bases, YGDEY’s effects on the intracellular localization of p65 could be due to the peptide’s antioxidant activity.

### 3.7. Regulatory Effect of YGDEY on MAPK Cell Signal Transduction

**Figure 7:**
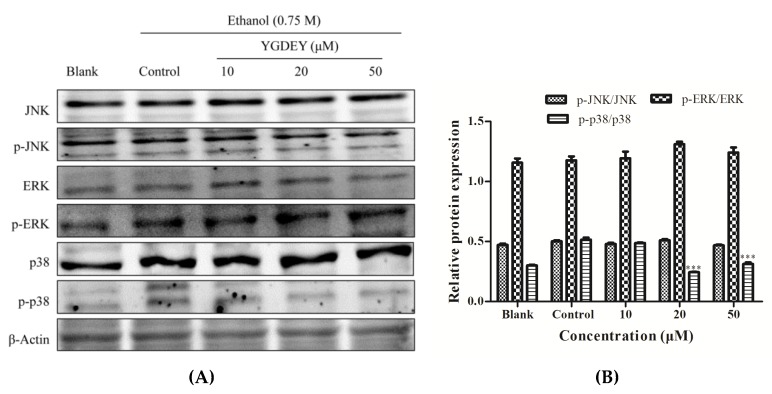
(**A**) Effect of YGDEY on the MAPK signaling pathway in HepG2 cells. Cells were pretreated with YGDEY (10, 20, and 50 μM) for 24 h before induction by 0.75 M ethanol for 2 h. β-Actin was used as an internal control. (**B**) Relative protein expression was normalized. Data are shown as means ± SD (*n* = 3). *** compared with the control group, *p* < 0.001.

This study investigated the effect of YGDEY on the MAPK pathway, particularly with regard to the levels of JNK, ERK, and p38. As depicted in Figure 7, ethanol treatment resulted in a significant dose-dependent increase in the expression of p-p38, while p-JNK and p-ERK showed no obvious changes. The level of p-p38 decreased with YGDEY treatment. Our results show that YGDEY inhibited the phosphorylation of p38 in the MAPK signaling pathway.

### 3.8. DNA Damage Analysis

**Figure 8:**
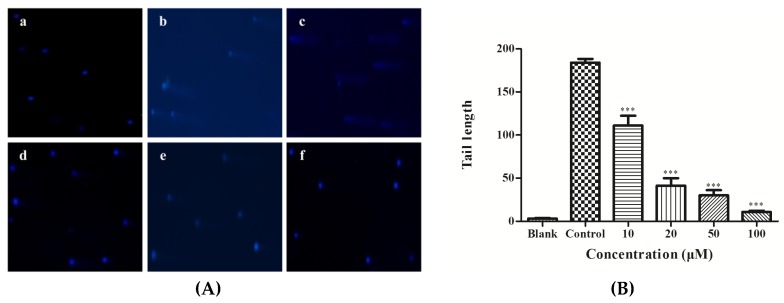
(**A**) Comet assay of (a) the blank group; (b) the control group; (c) 10, (d) 20, (e) 50, and (f) 100 μM of YGDEY for 24 h prior to ethanol treatment for 2 h followed by staining with DAPI. Images were obtained using an inverted fluorescence microscope with blue fluorescence (magnification: 10×). (**B**) Tail lengths of the comets were analyzed. Data are shown as means ± SD (*n* = 3). *** compared with the control group, *p* < 0.001.

As depicted in Figure 8A, there were no visible comets in the blank group. On the other hand, the control group—HepG2 cells induced by 0.75 M ethanol without YGDEY pretreatment—showed significant comets. The lengths of the comet tails decreased with increasing concentrations of YGDEY (Figure 8B). These results indicate that YGDEY reduced the amount of alcohol-induced DNA damage in HepG2 cells.

### 3.9. Molecular Docking Analysis

**Figure 9:**
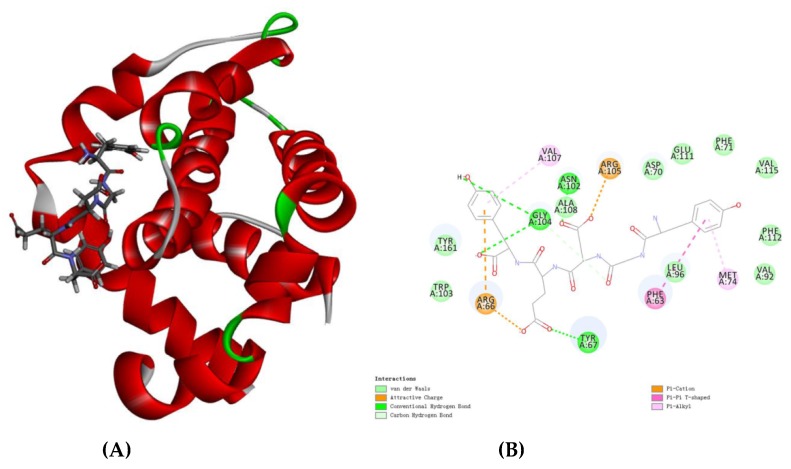
(**A**) The optimal docking structure of YGDEY and bcl-2; (**B**) 2D model of the interaction between YGDEY with the active site of bcl-2. YGDEY is represented by lines, and hydrogen bonds are shown with green dashed lines.

Bcl-2 is an anti-apoptosis protein [32]. To further support the results of the western blot analysis, the molecular docking method was carried out. There are 10 docking methods between YGDEY and bcl-2 in Table 1. According to -CDOCKER_INTERACTION_ENERGY, the first conformation was selected. As shown in Figure 9A, this is the optimal docking structure. Three hydrogen bonds (Tyr67, Asn102, and Gly104) form in the interaction between YGDEY and bcl-2 (Figure 9B). Furthermore, YGDEY forms a π–π interaction between the benzene ring and Phe63 of the bcl-2 protein (Figure 9B). The simulation results are consistent with the remarkable increase in the expression of bcl-2 in YGDEY-treated cells compared with the control group. The results indicate that YGDEY may be associated with the activity of bcl-2.

## 4. Discussion

The aim of this study was to observe the effects of ethanol and YGDEY treatment on HepG2 cells as a means to understand the hepatotoxicity of ethanol and the protective effect of YGDEY. Cellular viability is the most essential index in a cytotoxicity test [33]. The MTT assay revealed that ethanol treatment decreased cell viability (Figure 1B), a result that is similar to previous findings on HepG2 cells [34]. YGDEY (10, 20, 50, and 100 μM) had no obvious impact on HepG2 cell viability and had protective effects against ethanol-induced damage in HepG2 cells (Figure 1A,C).

Oxidative stress is the result of an imbalance between reactive oxidative species (ROS) and the antioxidant defense system in the body [23]. On the one hand, ROS are much more reactive than molecular oxygen [35]. It has been reported that a high rate of ROS generation can cause severe damage to DNA, the cell membrane, and proteins, ultimately leading to cell death [36]. Thus, ROS are considered to be the most critical markers of oxidative stress. Antioxidant systems comprise enzymatic and non-enzymatic mechanisms. The complex antioxidant system of cells mainly encompasses superoxide dismutase (SOD) and catalase (CAT), and the non-enzymatic antioxidants include glutathione (GSH) and vitamin C [37]. These molecules are responsible for protecting cells from the harmful effects of ROS. SOD is a key component of the antioxidant system: It is the first line of defense against oxidative stress [38]. After the dismutation of superoxide to H_2_O_2_ and O_2_ by this enzyme, CAT decomposes H_2_O_2_ to O_2_ and H_2_O. GSH acts as the second-line defense against oxidative damage [39] and plays a unique role in free radical scavenging [40]. GSH protects most tissues and cell lines from injury by oxidants and reactive electrophiles [41]. It has been established that cellular oxidative stress is often preceded by the depletion of intracellular GSH [42]. The enzyme GGT is derived from the plasma membrane of hepatocytes and is highly predictive of ALD [43]. Its function is related to the cellular uptake of amino acids [43]. In this study, YGDEY not only suppressed the overproduction of ROS but also enhanced the activities of SOD and GSH while decreasing the activity of GGT (Figure 2 and Figure 3). The restoration of SOD and GSH activity ameliorates alcohol-induced hepatotoxicity by reducing oxidative stress. These results are consistent with previous studies. For instance, LSGYGP from fish skin gelatin hydrolysates protected intercellular SOD activities from UVB damage in a dose-dependent manner [25]. Fish skin gelatin hydrolysate, produced by ginger powder, induced GSH synthesis to prevent hydrogen peroxide-induced intestinal oxidative stress [44]. VCSC and CAAP peptides from flounder fish (*Paralichthys olivaceus*) reduced intracellular ROS accumulation [45]. Our data from a model of alcohol-induced HepG2 cell damage suggest that the antioxidative activities of YGDEY are involved in its hepatoprotective effect.

The important role of oxidative stress in apoptosis has been well acknowledged [46]. Alcohol intake triggers ROS accumulation in liver cells, and overproduction of ROS induces cell death by apoptosis or necrosis. Apoptosis, the major form of controlled cell death [47], is the gene-controlled ordered death of a cell autonomously to maintain the stability of the inner environment [48]. Apoptosis is regulated by various molecules, such as the bcl-2 family proteins and the cysteinyl aspartate-specific proteinase (caspase) family [49,50]. The bcl-2 family can be classified into anti-apoptotic proteins (bcl-2 and bcl-x_L_) and pro-apoptotic proteins (bax, bak, and bad) [51]. High expression of bcl-2 can inhibit apoptosis, whereas high expression of bax can promote apoptosis [52]. The ratio of bax/bcl-2 determines whether apoptosis occurs and indicates a cell’s susceptibility to an apoptotic stimulus. The caspase family is broadly grouped into initiators (caspase-8 and caspase-9) and executioners (caspase-3 and caspase-7) in apoptosis [33]. In particular, caspase-3 is a frequent downstream effector caspase that catalyzes the cleavage of numerous vital cellular proteins [53]. The activation of caspase-3 is generally considered to be one of the most commonly manifested characteristics of the apoptosis stage for many cells [54]. The western blot results reported herein show that ethanol treatment increased the expressions of bax and cleaved-caspase-3 (c-caspase-3) compared with the blank group, but it decreased the level of bcl-2 (Figure 4A). YGDEY could markedly reduce the expressions of bax and c-caspase-3 and enhance the level of bcl-2 (Figure 4A). In addition, YGDEY prominently decreased the ratio of bax/bcl-2 and c-caspase-3/procaspase-3 compared with the control group (Figure 4B,C). These results are similar to those of a previous study [55]. However, the mechanism underlying the anti-apoptotic effect of YGDEY is still unclear and should be further studied.

Akt kinase can be activated by excessive ROS production [56]. Many studies have indicated that Akt pathways play a pivotal role in anti-apoptotic effects [57]. Nuclear factor-κB (NF-κB), consisting of the proteins p50, p65, and IκB, has been implicated in the control of apoptosis and autophagy [49]. In non-stimulated cells, NF-κB is located in the cytoplasm. Extracellular stimuli cause the rapid phosphorylation of IκB and its subsequent degradation, thus exposing the nuclear localization sequence on the p50–p65 heterodimer [58]. The p65 protein is then phosphorylated, leading to nuclear translocation. The data reveal that ethanol treatment increased the phosphorylation of Akt, IκB-α, and p65 in HepG2 cells. YGDEY significantly inhibited the phosphorylation of p65 via the activation of IκB-α and promoted the lessened the nuclear translocation of p65 (Figure 5 and Figure 6).

Mitogen-activated protein kinases (MAPKs) comprise the extracellular signal-related kinases (ERKs), c-Jun NH2-terminal kinases (JNKs), and the stress-activated p38 kinases [59]. The activation of MAPKs plays an important role in various physiological processes, such as cell proliferation, differentiation, apoptosis, autophagy, and DNA damage repair [60]. ERK is closely involved in cell growth and survival, while JNK and p38 mostly regulate the stress response and apoptosis [54]. It has been verified that ROS can activate MAPK signaling pathways, and, in turn, scavenging ROS can deactivate MAPK signal transduction pathways [60]. Increasing ROS levels often activates JNK and p38 proteins [61]. In the present study, the western blot results suggest that ethanol treatment activated the MAPK (p38) pathway, which resulted in HepG2 cell apoptosis. YGDEY downregulated the expression of p-p38 in ethanol-treated HepG2 cells (Figure 7). These data indicate that the p38 signaling pathway plays a central role in the preventive effects of YGDEY in HepG2 cells by promoting the anti-apoptotic response.

ROS tend to disrupt the stability of the DNA molecule [62]. The comet assay detects DNA damage to some extent in single cells and is one of the most utilized methods for this purpose [63]. The length of the comet tail represents the extent of DNA damage [6]. Long comet tail lengths were observed in the control group compared with the blank group (Figure 8), indicating that ethanol induced significant DNA damage in HepG2 cells. This may occur as a result of oxidative damage [64]. YGDEY treatment decreased DNA damage in HepG2 cells. Furthermore, the lengths of the comet tails decreased with increasing concentrations of YGDEY. These comet assay results shed new light on DNA damage levels in alcohol-treated HepG2 cells.

Additionally, due to strong hydrogen bonding (Figure 9B), YGDEY has a strong affinity toward bcl-2. The results of the molecular docking assay indicate that YGDEY may directly bind to bcl-2 to exert its biological activities.

It is well known that the antioxidant activity of a peptide is related to molecular size. A series of studies concluded that antioxidant peptides often contain fewer than 20 amino acid residues and can cross the intestinal barrier to exert their biological effects [65]. The molecular weights of these peptides are not more than 1500 Da. The peptide studied here (YGDEY, Tyr-Gly-Asp-Glu-Tyr) is 645.62 Da, which is consistent with the fact that an antioxidant peptide has a lower molecular weight. Moreover, amino acid compositions and sequences are also essential in a peptide with antioxidant activity. Hydrophobic and aromatic amino acids are correlated with the antioxidant properties of the peptides [17]. For instance, Tyr delivers a proton to suppress free radical generation. Ala, Val, Pro, Trp, Met, Pro, and Gly may contribute to antioxidative activity [45]. The phenomena observed in this study may be caused by the peptide’s hydrophobic amino acid (Gly) and aromatic amino acid (Tyr). These findings could be used to better understand the physiological effects of YGDEY and potentially use it in foods and pharmaceuticals.

## 5. Conclusions

In conclusion, YGDEY has significant protective effects against alcohol-induced hepatotoxicity in HepG2 cells. The present study demonstrates that YGDEY reduces ethanol-induced ROS production, DNA damage, Akt activation, p65 activation, and p38 activation, and inhibits alcohol-mediated apoptosis. These results reveal that YGDEY might be an important and novel hepatoprotective agent against ethanol-treated HepG2 cell damage via the decreased activation of Akt/NF-κB/MAPK signaling pathways. Therefore, YGDEY may have great potential for use as an active ingredient in functional foods and food supplements.

## Figures and Tables

**Table 1:** Methods of docking between bcl-2 and YGDEY.

Number	Receptor	Ligand	-CDOCKER_INTERACTION_ENERGY (kcal/mol)
1			71.4138
2			66.7418
3			66.3802
4			65.9307
5	bcl-2	YGDEY	65.7639
6			61.4377
7			61.2073
8			60.5086
9			60.1290
10			58.2131
